# Synthesis, Biological Activity and In Silico Pharmacokinetic Prediction of a New 2-Thioxo-Imidazoldidin-4-One of Primaquine

**DOI:** 10.3390/ph14030196

**Published:** 2021-02-27

**Authors:** Mariana Pereira, Guy Caljon, Maria João Gouveia, Louis Maes, Nuno Vale

**Affiliations:** 1 OncoPharma Research Group, Center for Health Technology and Services Research (CINTESIS), Rua Dr. Plácido da Costa, 4200-450 Porto, Portugal; mariana.m.pereira2097@gmail.com (M.P.); mariajoaogouveia@gmail.com (M.J.G.); 2 Laboratory of Parasitology, Microbiology and Hygiene (LMPH), Faculty of Pharmaceutical, Biomedical and Veterinary Sciences, University of Antwerp, Universiteiplein 1, 2610 Antwerp, Belgium; guy.caljon@uantwerpen.be (G.C.); louis.maes@uantwerpen.be (L.M.); 3 Faculty of Medicine, University of Porto, Al. Prof. Hernâni Monteiro, 4200-319 Porto, Portugal

**Keywords:** primaquine, 2-thioxo-imidazolidin-4-one, antitrypanosomal activity, in silico PK

## Abstract

The discovery of novel antiparasitic drugs for neglected tropical diseases (NTDs) constitutes a global urgency and requires a range of innovative strategies to ensure a sustainable pipeline of lead compounds. Thus far, primaquine (PQ) is the only transmission-blocking antimalarial that is clinically available, displaying marked activity against gametocytes of all causative species of human malaria (*Plasmodium* spp.). Chagas disease, caused by *Trypanosoma cruzi*, is another PQ-sensitive illness besides malaria. One of the major drawbacks of PQ is its metabolism into carboxyprimaquine (CPQ), which is less active than the parent drug. In this study, we developed different synthetic pathways to confer N-protection to PQ through introduction of thioxo-imidazolidin-4-one. The introduction of this group prevents the formation of CPQ, counteracting one major drawback of the parent drug. After that, we evaluated the potential biological activity of the novel 2-thioxo-imidazolidin-4-one derivative of PQ, which showed relevant in vitro activity against *Trypanosoma cruzi* (IC_50_ 1.4 μM) compared to PQ (IC_50_ 1.7 μM) and the reference drug benznidazole (IC_50_ 1.6 μM). Noting its acceptable pharmacokinetic profile, this PQ conjugate may be a potential scaffold for novel drug exploration against Chagas disease.

## 1. Introduction

Protozoan parasites that infect humans represent a significant threat to health, causing more than a billion deaths annually, particularly in developing countries. The World Health Organization (WHO) has expressed great concern for these neglected tropical diseases (NTDs) [1]. A subset of life-threatening NTDs includes leishmaniasis, malaria, sleeping sickness and Chagas disease, among others. Difficulties associated with controlling the sources of infection, the high cost of treatment/prevention and poor compliance contribute to the propagation and difficulty of eliminating these NTDs [2]. The global burden is exacerbated by the lack of licensed vaccines for these diseases. Treatment and prophylaxis have been dependent on drugs, many of which have become less effective, necessitating the search for replacements. Moreover, the activity of most drugs has decreased due to drug resistance developed by parasites [3].

**Figure 1:**
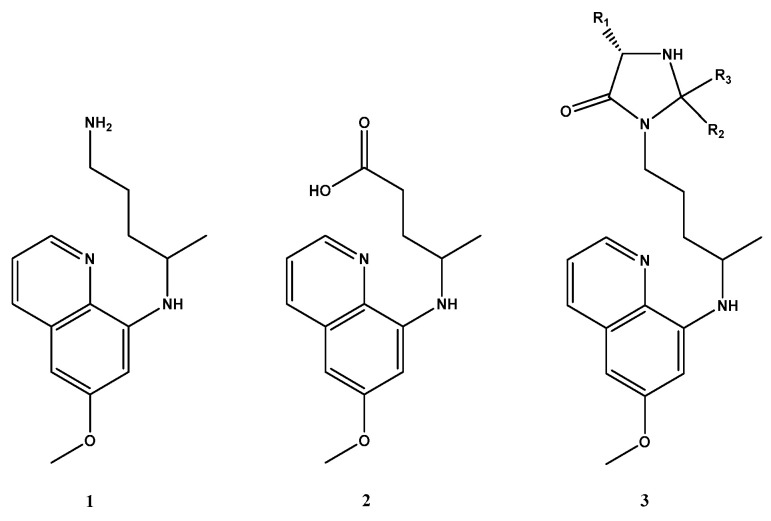
Chemical structures of primaquine (PQ) (**1**), carboxyprimaquine (CPQ) (**2**) and imidazolidin-4-one group (Imd) (**3**).

Primaquine (PQ, **1**, Figure 1) is an antimalarial from the group of 8-aminoquinolines, developed in the United States of America (USA) to prevent American troops infected during the Korean War in the 1950s from relapsing [4]. PQ can be used as (i) a primary prophylactic to destroy *Plasmodium ovale* and *P. vivax* before they can cause the disease; (ii) a radical cure after the onset of disease in combination with chloroquine; and (iii) terminal prophylaxis to prevent relapse due to hypnozoites in the liver [5]. It is thought that the mode of action of PQ is related either to interference with the mitochondria and the electron respiratory chain or to the production of intracellular oxidants, but this mechanism is not yet completely understood [6]. These antimalarial effects are attributed mainly to reactive metabolites of the parent drug resulting from hepatic biotransformation [7]. In terms of pharmacokinetics, PQ is absorbed from the gastrointestinal tract after oral administration with a blood peak level around 1.5 h. It is quickly distributed throughout the body and metabolized into carboxyprimaquine (CPQ, **2**, Figure 1), its main metabolite. CPQ is substantially less active than the parent drug, with only 1% of total dosage being excreted as unmetabolized PQ through urine [8,9]. Metabolism of PQ mainly occurs in the liver, which is important for the antimalarial effect. Excretion occurs primarily through feces [10].

The antiparasitic activity of PQ can be directly related to its metabolism by CYP_2D6_. It has been shown that inhibition of this enzyme leads to the accumulation of PQ in the organism and decreases the levels of the metabolites that are responsible for antimalarial activity [11,12,13]. In humans, CYP_2D6_ is polymorphic. A study of the CYP_2D6_ phenotype in soldiers demonstrated that those with poor or intermediate metabolism only produced minimal amounts of 5,6-ortho-quinone, a stable metabolite thought to be responsible for PQ activity [14]. Hence, it is important to adjust the PQ dosage according to CYP_2D6_ phenotype to boost drug efficiency and decrease possible toxic effects.

Generally, PQ is considered to be a safe drug. Nevertheless, the administration of PQ damages erythrocytes due to free-radical oxidative stress that is exacerbated in patients with glucose-6-phosphate dehydrogenase (G6PD) deficiency [11]. The combination of oxidant hemolysis with G6Pd can ultimately culminate in acute hemolytic anemia. Therefore, it is of utmost importance to confirm the existence of G6PD deficiency before PQ administration [12]. PQ is metabolized in phase I by the main catalytic enzyme monoamine oxidase (MAO-A) and in phase II by enzymes of the CYP_450_ family, namely, CYP_2D6_ [15]. As already mentioned, the main metabolite of PQ in human plasma is CPQ, which has a slower clearance and lower distribution [16]. The formation of CPQ is mainly associated with phase-I metabolism by MAO-A that results in deamination and formation of an aldehyde that is potentially reduced by formic acid, forming a PQ alcohol as precursor to CPQ [12]. Despite oxidative deamination mainly associated with MAO-A, CYP_450_ can also catalyze this reaction, although to a lesser extent, and can be associated with the broader distribution of MAO-A in the organs while CYP_450_ is mainly restricted to the liver [14]. This major metabolite is inactive and is not responsible for the antimalarial effects of PQ [7]. PQ can also be oxidized by CYP_2D6_ into several hydroxyl metabolites [15,16,17,18] that can in turn be oxidized into quinone-imines, generating H_2_O_2_ as a byproduct. Quinone-imines can be reduced into the hydroxyl metabolites by CYP_450_ NADPH oxidoreductase, forming a redox cycle that leads to accumulation of H_2_O_2_ [15]. The quinoline core of PQ, 5-hydroxyprimaquine (that converts into 5,6-ortho-quinone) is the most unstable metabolite and is thought to be responsible for activation of redox cycling. This core may confer the antimalarial activity of PQ but also its hemolytic toxicity [19]. A third established pathway is a direct phase-II metabolism by conjugation of PQ with glucose, acetate, carbamate or glucuronide. The N-carbamoyl glucuronide PQ conjugate is an example of that reaction and is formed when carbamoyltransferases induce N-carbamoylation of PQ followed by conjugation of glucuronide through UDP-glucuronosyltransferases [20].

Our research group recently developed a new synthetic pathway to protect metabolization of PQ into CPQ through the introduction of the imidazolidin-4-one group (Imd, **3**, Figure 1). These derivatives inhibited the sporogonic cycle of *P. berghei*, affecting the appearance of oocysts in the midgut of mosquitoes [21]. Our compounds were also screened for their in vitro activity against *Pneumocystis carinii* and the *P. falciparum* W2 strain. Besides the elevated antifungal activity against *P. carinii,* the derivatives were also the most active antiplasmodial agents. One imidazolidin-4-one was slightly more active than PQ [22], providing an 8-aminoquinoline with increased stability and resistance to metabolic inactivation.

Even through **1** is not being clinically used against Chagas disease, both **1** and its 2-methylprimaquine derivative were reported to be almost four times as effective as nifurtimox in the mouse model [23]. One of the hypotheses for the anti-trypanosomal activity of **1** and related 8-aminoquinolines (8-AQ) relies on the metabolic formation of free radicals that increase the oxidative stress on *T. cruzi* [24].

Herein, we describe several synthetic routes for new PQ derivatives that incorporate 2-thioxo-imidazolidin-4-one to prevent the formation of CPQ. In addition, in vitro profiling for antiprotozoal and antibacterial activity and in silico prediction of pharmacokinetic (PK) properties were included.

## 2. Results and Discussion

NTDs such as malaria, visceral leishmaniasis, Chagas disease and sleeping sickness affect millions of people in developing countries. Although these diseases have profound health and socio-economic impacts, the funding available for novel control tools and innovative drug research and development remains limited. Consequently, the majority of chemotherapeutic treatments for NTDs mostly rely on single drugs that cause several adverse effects and are marginally effective [25]. These facts emphasize the great need to develop new treatment strategies that include extending the usefulness of existing drugs by generating new formulations, drug combinations/dose regimens and drug repurposing.

PQ (**1**) is the most representative 8-aminoquinoline antimalarial and is still the only clinically available transmission-blocking drug with marked activity against gametocytes of all species of human malaria. However, PQ is often associated with adverse effects due to toxic metabolites that are considered directly responsible for complications such as hemolytic anemia. Adverse effects are further amplified by the fact that PQ must be repeatedly administered at high doses due to its limited oral bioavailability [6,26]. Although PQ is mostly used for malaria, there are other PQ-sensitive illnesses such as Chagas disease.

**Figure 2:**
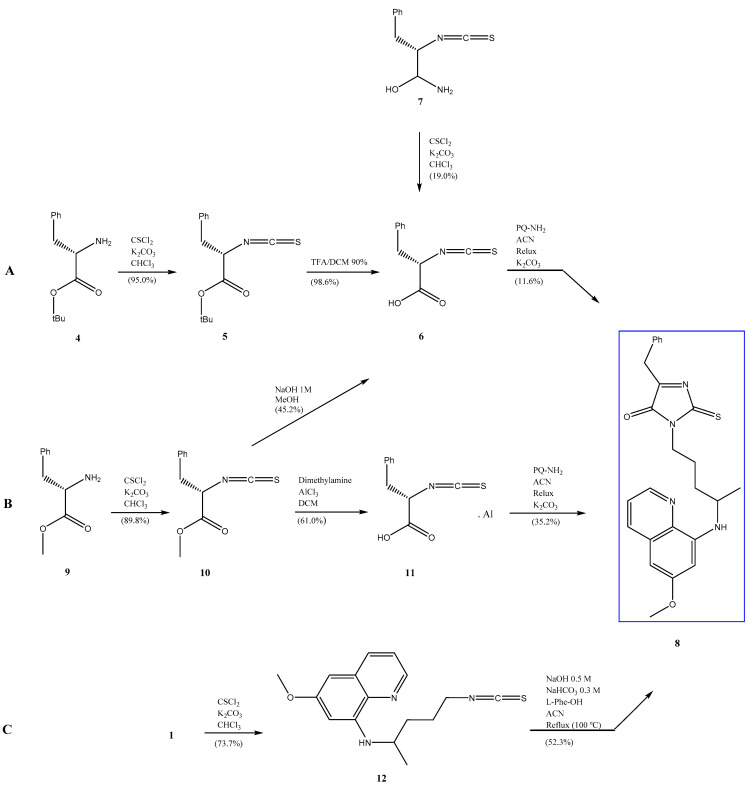
Different methodologies developed to obtain the synthesis of 2-thioxo-imidazolidin-4-one of PQ (**8**), using different starting reagents: (**A**) *L*-Phenylalanine *tert*-butyl ester, (**B**) *L*-Phenylalanine methyl ester and (**C**) PQ.

Through different synthetic pathways (Figure 2), we developed a 2-thioxo-imidazolin-4-one PQ derivative (**8**) that may prevent the formation of one major metabolite of **1** through N-protection with a 2-thioxo-imidazolidin-4-one group. Synthetic route C using **1** as starting material was the most rapid (only three steps) while route B achieved the best yield (52.2%).

**Figure 3:**
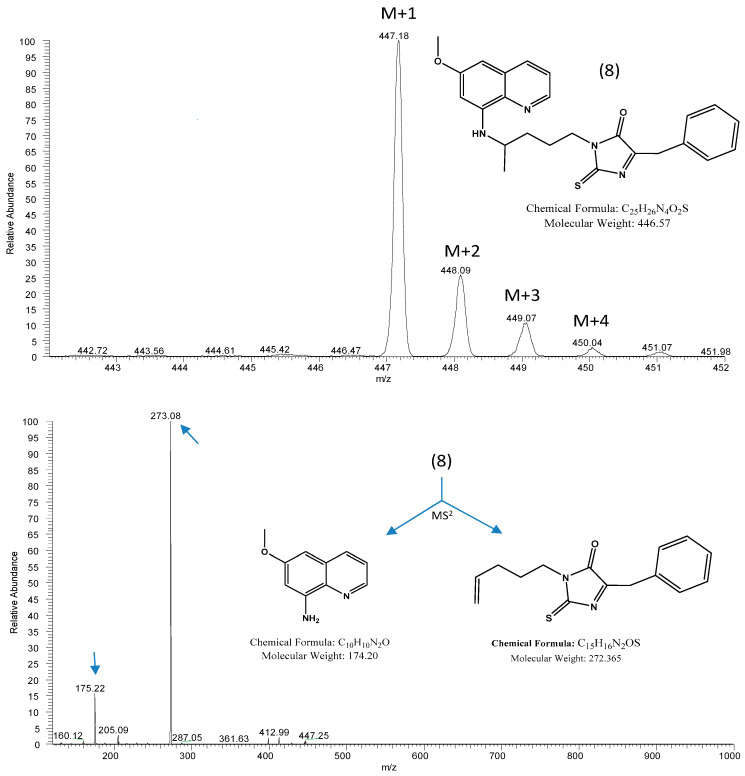
Product ion spectrum (**top**) with [M + H]^+^ (or M + 1) and +2, +3 and +4 isotope peak of 2-thioxo-imidazolidin-4-one derivative of PQ (**8**). Proposed fragmentation scheme of **8** (**down**).

The hydrolyses of **8** and PQ were evaluated in human plasma (Table 1). Considering the proximity of values for PQ with reference values in this study, we observed a high level of stability after 2-thioxo-imidazolidin-4-one formation. Compound **8** was also stable in pH 7.4 buffer solution and hydrolyzed to the corresponding parental drug, with half-lives ranging up to 72 h, and is thus a potential new derivative of PQ that can prevent oxidative deamination to the inactive metabolite CPQ. This stability is in accordance with the fragmentation data obtained by mass spectrometry (Figure 3), in which the pentagonal ring has stability and the capacity to protect the terminal amine of the aliphatic chain of the initial drug and thus prevent the formation of the inactive derivative (CPQ).

PQ and **8** were evaluated in integrated screening of broad spectrum activity for their antiprotozoal (antileishmanial, antiplasmodial and antitrypanosomal) and antibacterial potential (Table 2). The biological activity of **8** was compared with **1** and respective reference drugs. For all assays, the relatively highest activity was found against *T. cruzi* with an IC_50_ of 1.4 μM, which is marginally better than benznidazole (1.6 μM) and PQ (1.7 μM). In agreement with the reported efficacy of **1** against *T. cruzi* [27], the new derivatives of **1** might be useful against other NTDs besides malaria although **1** is not clinically used for Chagas disease. Cytotoxicity was evaluated in fetal human lung fibroblasts (MRC-5 cell line). Acceptable cytotoxicity was noted for both **1** and **8** (IC_50_ > 30 μM). The introduction of the 2-thioxo-imidazolidin-4-one group may protect against the formation of inactive metabolites of PQ and increase selective activity against *T. cruzi*. Compound **8** showed moderate activity against the other protozoans and was inactive against *S. aureus* (IC_50_ > 64 μM).

**Figure 4:**
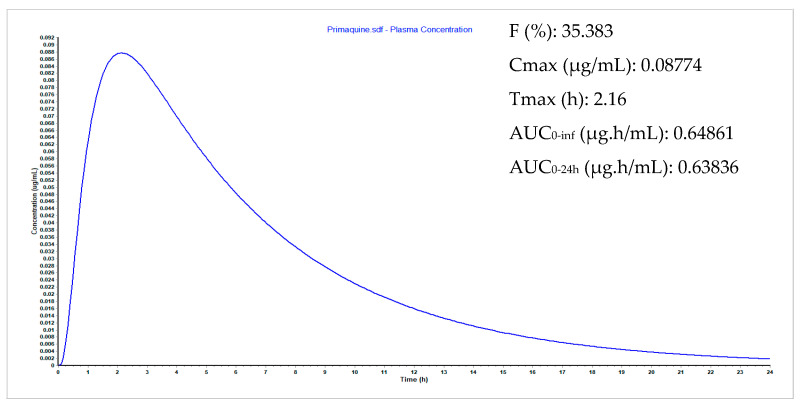

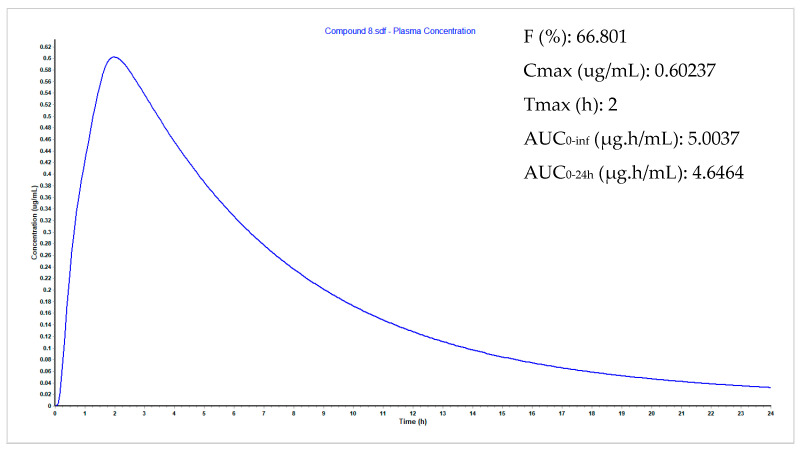
Plasma concentration-time curve of PQ (**1**) and 2-thioxo-imidazolidin-4-one derivative of PQ (**8**) as simulated using GastroPlus™. Values shown in the upper right corner of each image refer to the population simulation results of PK parameters F%, maximum concentration (C_max_), time of maximum concentration (Tmax), AUC_0-inf_ and AUC_0–24h_.

To predict oral exposure, a physiologically based oral absorption model was developed using GastroPlus™ software [28]. The compound properties were defined by in silico simulation using chemical structure and reference formulations from the software. The maximum concentration (C_max_), area under the curve (AUC), bioavailability (F) and time of maximum concentration (T_max_) were derived by simulation adopting the same method and considering the predictions at the same time points. According to the predicted plasma concentration-time curves (Figure 4), **8** showed good performance for all PK parameters. The bioavailability of 67% for **8**, compared to 35% for PQ, is relevant, as is its shorter T_max_ and higher C_max_. These simulated data (Figure 4) can be potential indicators that incorporation of thioxo-imidazolidin-4-one may improve the PK profile of **8**.

Altogether the results obtained in this study indicate that the protection of PQ from being metabolized could be relevant to increasing its activity not only against *Plasmodium* species but also other diseases such as Chagas disease. On the other hand, this synthetic pathway results in derivatives with improved pharmacokinetic profiles. Further studies including in vivo assays should be performed. Thus far, the protection of PQ with other chemical groups should be pursued for the development of novel derivatives that can be used not only against malaria but also other diseases, including Chagas disease.

## 3. Materials and Methods

### 3.1. Chemicals

The new 2-thioxo-imidazolidin-4-one derivative of PQ (**8**, Figure 1) was synthesized through different pathways (Figure 2). PQ (**1**) and all reagents were obtained from Sigma Aldrich/Merck (Algés, Portugal). All intermediate compounds were purified and characterized by ESI-MS, ^1^H-NMR and ^13^C-NMR (data not shown).

The first step (Figure 2) in route C involves the formation of isothiocyanate of PQ (**12**) and then the making of a mixture of PQ isothiocyanate (1 eq.), amino acid (1 eq.), and NaOH 0.5 M with acetonitrile (ACN) as solvent. The mixture was heated under reflux for 30 min. Next, 1.1 eq. of NaHCO_3_ 0.3 M was added, mixed and heated under reflux for 2.5 h. After evaporation of the organic solvent, liquid–liquid extraction was carried out with dichloromethane and 10% NaHCO_3_. The organic phases were concentrated, and a precipitate was obtained that, after drying, turned yellow. The compound (**8**) was characterized by mass spectrometry using electrospray ionization (ESI) (for **8**, Figure 3) and the nuclear magnetic resonance of ^1^H and ^13^C: δH (CDCl_3_, 400 MHz) 8.50 (m, 1H); 7.91 (d, 1H, J = 8.16 Hz); 7.21–7.25 (m, 5H); 6.31 (d, 1H, J = 2.45 Hz); 6.25 (d, 1H, J = 2.44 Hz); 5.99 (dd, 1H, J = 8.20, 5.60 Hz); 3.87 (s, 3H); 3.75 (t, 1H, J = 5.39 Hz); 3.84–3.71 (m, 2H); 2.88 (d, 2H, J = 5.20 Hz); 2.74 (m, 1H); 1.14 (s, 3H). δC (CDCl_3_, 100 MHz) 183.7; 166.9; 159.4; 157.3; 145.0; 144.3; 136.9; 135.3; 134.7; 129.6; 129.4; 128.6; 128.2; 126.6; 121.6; 97.7; 96.5; 58.8; 55.1; 53.9; 35.4; 28.6; 22.4; 20.9.

### 3.2. Hydrolysis in Aqueous Solution

The rates of hydrolyses of compound **8** were determined in pH 7.4 phosphate saline buffer, at 37 °C. A 10 μL aliquot of a 10^−2^ M stock solution of substrate in acetonitrile was added to 10 mL of the appropriate thermostated buffer solution. At regular intervals, samples of the reaction mixture were analyzed by HPLC (the analytical conditions were reported by our group using imidazolin-4-ones of PQ [21]). All reactions followed first-order kinetics over four half-lives.

### 3.3. Biological Assay

The integrated panel of microbial screens and standard screening methodologies were adopted as previously described [29,30]. All assays were performed at the Laboratory of Microbiology, Parasitology and Hygiene at the University of Antwerp, Belgium. Compounds **1**, **8** and reference drugs were tested in triplicate at five concentrations (64, 16, 4, 1 and 0.25 μM) to establish a full dose-titration and determination of IC_50_ values. The in-test concentration of DMSO (used to prepare compound stock solutions) did not exceed 0.5%. Selectivity of action was assessed by simultaneous evaluation of cytotoxicity on a fibroblast (MRC-5) cell line.

### 3.4. Antitrypanosomal Activity

*Trypanosoma brucei* Squib-427 strain (suramin-sensitive) was cultured at 37 °C and 5% CO_2_ in Hirumi-9 medium supplemented with 10% fetal calf serum (FCS). About 1.5 × 10^4^ trypomastigotes were added to each well, and parasite growth was assessed after 72 h at 37 °C by adding resazurin. For Chagas disease, *T. cruzi* Tulahuen CL2 (benznidazole-sensitive) was maintained on MRC-5 cells in minimal essential medium (MEM) supplemented with 20 mM L-glutamine, 16.5 mM sodium hydrogen carbonate and 5% FCS. In the assay, 4 × 10^3^ MRC-5 cells and 4 × 10^4^ parasites were added to each well. After incubation at 37 °C for 7 days, parasite growth was assessed by adding the β-galactosidase substrate chlorophenol red and β-D-galactopyranoside. The color reaction was read at 540 nm after 4 h incubation at 37 °C. The results were expressed as percentage (%) reduction in parasite burdens compared to control wells, and an IC_50_ was calculated. Suramin and benznidazole were included as reference drugs against *T. brucei* and *T. cruzi*, respectively.

### 3.5. Antiplasmodial Activity

Chloroquine-resistant *P. falciparum* K1-strain was grown in human erythrocytes O^+^ at 37 °C under a low oxygen atmosphere (3% O_2_, 4% CO_2_ and 93% N_2_) in RPMI-1640 medium supplemented with 10% human serum. Infected human red blood cells (200 μL, 1% parasitaemia and 2% hematocrit) were added to each well containing the prediluted compounds and incubated for 72 h. After incubation, test plates were frozen at −20 °C. After thawing, 20 μL of a 1/1 mixture of PES (phenazine ethosulphate, 0.1 mg/mL) and Nitro Blue Tetrazolium (NBT) (Grade III, 2 mg/mL) were added. Change in color was measured spectrophotometrically at 655 nm. The results were expressed as percentage reduction in parasitaemia compared to the control wells. Chloroquine was included as a reference drug.

### 3.6. Antileishmanial Activity

*Leishmania infantum* MHOM/MA(BE)/67 amastigotes were maintained in Golden hamsters, and spleen amastigotes were collected for preparation of the infection inoculum. Primary peritoneal mouse macrophages were used as host cells and were collected 2 days after peritoneal stimulation. Assays were performed in 96-well microtiter plates, with each well containing 10 µL of the compound dilutions together with 190 µL of macrophage/parasite inoculum. After 5 days, parasite burdens (mean number of amastigotes/macrophage) were microscopically assessed after Giemsa staining. The results were expressed as percentage reduction in parasite burden compared to untreated control wells and an IC_50_ was calculated. Amphotericin B (Fungizone^®^, Bristol-Myers Squibb, Brussels, Belgium) was included as a reference drug.

### 3.7. Antibacterial Activity

*Staphylococcus aureus* bacteria were cultured at 37 °C in Mueller-Hinton Broth (MHB) medium at 37 °C. Assays were performed in 96-well microtiter plates, with each well containing 10 μL of prediluted compound together with 190 μL of inoculum (5 × 10^3^ colony-forming units (CFU)/mL). After 17 h incubation, the bacterial viability was assessed fluorometrically by adding 10 µL resazurin per well for 30 min at 37 °C [30]. The results were expressed as percentage reduction in bacterial growth/viability compared to the control wells. Erythromycin was used as a reference drug.

### 3.8. Cytotoxicity Assay

MRC-5SV2 cells were cultivated in MEM supplemented with L-glutamine (20 mM), 16.5 mM sodium hydrogen carbonate and 5% FCS. For the assay, 10^4^ MRC-5 cells/well were seeded onto the test plates containing the prediluted compounds and incubated at 37 °C and 5% CO_2_ for 72 h. Cell viability was assessed fluorometrically 4 h after addition of resazurin. Fluorescence was measured (excitation 550 nm, emission 590 nm), and the results were expressed as percentage reduction in cell growth/viability compared to untreated controls. Tamoxifen was included as a cytotoxic reference compound.

### 3.9. Plasma Concentration Simulations

The software GastroPlus™ version 9.6 (Simulations Plus Inc., Lancester, CA, USA) was used to predict the plasma concentration of PQ (**1**) and the 2-thioxo-imidazolidin-4-one derivative of PQ (**8**). Input data consisted of values taken from literature for PQ, including but not limited to drug solubility, pKa, and partition coefficient LogP, as well as other parameters obtained using the ADMET Predictor™ (Absorption, Distribution, Metabolism, Excretion and Toxicity Predictor) (Simulations Plus, Lancaster, CA, USA) module in GastroPlus™. Using the same model, parameters for compound **8** were obtained from chemical structure and simulated plasma concentrations to obtain PK parameters, also developed in other projects [31,32] and by our research group [33,34].

## 4. Conclusions

The new 2-thioxo-imidazolidin-4-one derivative of PQ (**8**) was slightly more effective against *T. cruzi* than the parent PQ and the reference drug benznidazole. In human plasma, compound **8** was stable, with PQ being released only slowly over longer incubation periods. Simulated PK parameters further support that **8** can be a useful scaffold for further studies on new drug candidates for Chagas disease.

## Figures and Tables

**Table 1:** In vitro stability studies of PQ and 2-thioxo-imidazolidin-4-one derivative (**8**) of PQ.

	Human Plasma		PBS
Compound	t_1/2_ (h) ^a^	t_1/2_ (h) ^b^	t_1/2_ (h) ^c^
**PQ**	3.46	3.70	ND
**8**	3.86	ND	>72

^a^ This study, using GastroPlus; ^b^ As PQ diphosphate; from Food and Drug Administration (FDA)/Drug Bank; ^c^ Hydrolyses in pH 7.4 phosphate saline buffer (PBS) at 37 °C. ND, Not Determined.

**Table 2:** In vitro antiprotozoal, antibacterial and cytotoxicity of PQ (**1**), 2-thioxo-imididazolidin-4-one of PQ (**8**) and respective reference drugs. IC_50_ values are expressed in μM.

Compound	*T. cruzi*	*T. brucei*	*P. falciparum*	*L. infantum*	*S. aureus*	MRC-5
**1**	1.7 ± 0.1	8.2 ± 0.4	2.2 ± 0.2	4.0 ± 0.5	>64.0	34.8 ± 1.8
**8**	1.4 ± 0.05	8.4 ± 0.6	10.2 ± 0.5	20.3 ± 1.1	>64.0	30.1 ± 1.6
Benznidazole	1.6 ± 0.03	-	-	-	-	-
Suramin	-	0.04 ± 0.02	-	-	-	-
Chloroquine	-	-	0.2 ± 0.02	-	-	-
Amphotericin B	-	-	-	1.2 ± 0.1	-	-
Erythromycin	-	-	-	-	10.4 ± 0.7	-
Tamoxifen	-	-	-	-	-	10.8 ± 0.8

## Data Availability

Data sharing not applicable.
